# Phylogeography and population genetics of the white spotted eagle ray, *Aetobatus laticeps* Gill, 1865, in the Eastern Tropical Pacific

**DOI:** 10.1371/journal.pone.0349373

**Published:** 2026-05-18

**Authors:** Mateo Vinueza Guarderas, María de Lourdes Torres, María José Pozo Andrade, Adriana Cevallos, Javier Tovar-Ávila, Rossana Maguiño, Daniella Biffi, Carmen González, Sebastián Hernández, Diana A. Pazmiño

**Affiliations:** 1 Laboratorio de Biotecnología Vegetal, Colegio de Ciencias Biológicas y Ambientales, Universidad San Francisco de Quito, Quito, Pichincha, Ecuador; 2 Galápagos Science Center (GSC), Colegio de Ciencias Biológicas y Ambientales, Universidad San Francisco de Quito, Puerto Baquerizo Moreno, Galápagos, Ecuador; 3 Facultad de Ciencias del Mar, Universidad Laica Eloy Alfaro de Manabí (ULEAM), Manta, Ecuador; 4 Centro Regional de Investigaciones Acuícolas y Pesqueras (CRIAP), Instituto Mexicano de Investigación en Pesca y Acuacultura Sustentable (IMIPAS), Bahía de Banderas, Nayarit, México; 5 Fundación ecOceánica, San Borja, Lima, Perú; 6 Biomolecular Laboratory, Center for International Programmes and Sustainability Studies, Universidad LCI Veritas, San José, Costa Rica; 7 Sala de Colecciones, Facultad de Ciencias del Mar, Universidad Católica del Norte, Coquimbo, Elqui, Chile; 8 Pontificia Universidad Católica del Ecuador-Sede Manabí (PUCESM), Manta, Ecuador; 9 MigraMar, Olema, California, United States of America; University of Padova: Universita degli Studi di Padova, ITALY

## Abstract

The Eastern Tropical Pacific (ETP) Spotted Eagle Ray, *Aetobatus laticeps* Gill, 1865, is an understudied species with limited information on its ecology and conservation status. Only in the last 10 years has this lineage been re-described as a distinct species from the formerly widespread *Aetobatus narinari* species complex (Euphrasen, 1790) (ANSC). The sampling in the studies that divided the ANSC was not geographically balanced, with most individuals (50) collected from the Atlantic and only (5) from the ETP. Given the vast extent and environmental heterogeneity of this region, it is possible that a significant portion of the genus’s genetic variation, and possibly even the presence of undescribed species, is being overlooked. Because eagle rays have relatively low fecundity and are subject to targeted and incidental fisheries, they are highly susceptible to population declines. Consequently, elucidating the genetic diversity, distribution range, genetic variability and interconnectivity of this species is crucial to assess its conservation status correctly. Through a mixed genetic marker approach, we assessed ETP eagle rays’ phylogeny, genetic diversity and phylogeography. First, we confirmed that eagle rays in this region correspond to *A. laticeps.* Second, we found low levels of genetic variability across all markers, coupled with a high degree of geographic-genetic structure. Finally, our data suggested three potential mechanisms that could explain the population structure we encountered: a) Isolation by distance b) Isolation by philopatry, and c) Isolation by depth. To date, this is the first comprehensive study of the genetic diversity of eagle rays in the Eastern Tropical Pacific, shedding light on a species that has long been overlooked. Our findings suggest that this species is more susceptible to direct and bycatch fishing pressures, as well as other indirect human impacts. Therefore, we recommend a re-evaluation of the conservation status of this species.

## Introduction

Spotted eagle rays of the genus *Aetobatus* are benthopelagic mesopredators that primarily forage for crustaceans and mollusks in shallow coastal zones [1]. Like many other batoids, *Aetobatus* rays are ovoviviparous and exhibit relatively long gestation periods and small litter sizes. Parturition typically occurs in protected costal habitats like coral lagoons, rocky reefs and mangrove forests [1–4]. The Aetobatidae family, of which *Aetobatus* is the only representative genus [5], has recently undergone several taxonomic revisions, and is now presumed to be comprised of five species: *Aetobatus flagellum* (Bloch & Schnider, 1801), *Aetobatus narutobiei* White, Furumitsu & Yamaguchi, 2013, *Aetobatus narianari* (Euphrasen, 1790), *Aeotbauts ocellatus* (Kuhl, 1823), and *Aetobatus laticeps* Gill, 1865 [6–8]. Most of the species that now form part of the *Aetobatus* genus originated from the taxonomic reclassification of the white spotted eagle ray, *Aetobatus narinari* [6–8]. As with most species exhibiting a cosmopolitan distribution, *A. narinari* was long suspected to be an intricate cryptic species complex. Differences in the parasitic communities of geographically distant populations of *A. narinari* led researchers to question the species’ pantropical occurrence [6,9]. Only in the last decade, with the advent of molecular tools for species delimitation, has the *A. narinari* species complex (ANSC) been conclusively divided into at least three independently evolving taxa [i.e., species]: *A. narinari* in the central Atlantic Ocean, *A. ocellatus* in the Indian Ocean and Western Pacific, and *A. laticeps* in the Eastern Tropical Pacific from the Gulf of California in the North to Gulf of Guayaquil in the South [6,7]. Although a number of studies have concluded that populations of *A. narinari* off Southern Africa represent a distinct species from their Caribbean and western Atlantic counterparts and are more closely related to the Indo-Pacific A. ocellatus [6,7,10].

The phylogenetic divergence of the ANSC appears to have resulted from two speciation events: An initial split between the Indo-Pacific (*A. ocellatus*) and the Atlantic–Eastern Pacific (*A. narinari* and *A. laticeps*) lineages around 3.7 Mya, followed by a the separation between Atlantic and Eastern Pacific populations approximately 1.4 Mya [6,7]. Richards et al. [6] theorized that the closure of the Panama Isthmus formed a barrier that disconnected gene flow between the Atlantic and the Eastern Pacific populations of *A. laticeps* and *A. narinari’s* common ancestor. However, Sales et al. [7] questioned whether the Isthmus of Panama acted as a vicariant barrier during the late Pleistocene, as the most widely accepted estimate for its emergence predates the divergence between the Eastern Pacific and Atlantic eagle ray lineages by approximately two to four million years. Richards et al. [6] and Sales et al. [7] suggested that the most likely point of origin of the common ancestor of the ANSC was located in the Indo-Pacific, and that it later radiated westward towards the Atlantic and finally to the Eastern Pacific. This westward migration was probably facilitated by the east-to-west direction of equatorial oceanic currents generated by trade winds [11]. Trade wind–driven currents and the Pacific Walker circulation may also help explain why the ANSC did not directly reach the Eastern Pacific from its Indo-Pacific region of origin [7,11].

Extensive research has been conducted on *A. narinari* in the Atlantic and, to a lesser extent, on *A. ocellatus* in the Western Pacific, providing insights into their biology, behavior, genetic diversity, population structure, phylogeography, phylogenomics, and even their microbiomes [2–4,10,12–16]. For example, on a wide assessment of *A. narinari* eagle rays in the Gulf of Mexico, Sellas et al [2] found significant differentiation in both nuclear microsatellites and mitochondrial CYTB sequences, which could not be solely explained by the geographic distances between sampled populations. Strong philopatric tendencies accompanied by sex-biased dispersal have been documented for *A. narinari,* indicating a possible source for the genetic structuring observed in this and other species within this genus [3,13]. Similarly, Schluessel et al. [17], reported significant population structure, with limited gene flow between *A. ocellatus* from 5 localities in the central Indo-Pacific. Furthermore, the impact of directed and collateral human pressures on these species’ stocks has also been documented and assessed at multiple localities throughout their distributions [2,12,14,15,18–21]. Tagliafico et al. [18], found that the landings from targeted *A. narinari* fisheries in northeastern Venezuela presented a 2.6:1 female to male ratio across various size classes, and that many of the captured females were gravid. Considering this species’ low fecundity and long gestational terms, the differential removal of females represents a critical threat to the future viability of eagle ray populations. On the other hand, most aspects of the ecology of *A. laticeps,* with the exception of some behavioral, and non-invasive marking studies, remain largely unexplored [22–24]. To date, no study has comprehensively assessed the demographic trends, population genetic structure, or anthropogenic threats affecting *A. laticeps*. Although some information on its genetic diversity is available, existing studies are geographically limited and based on small, potentially biased sample sizes. For instance, the molecular studies that partitioned the ANSC (Richards et al. [6]; Sales et al. [7]) focused primarily on Atlantic and Western Pacific lineages and included only eight individuals from the ETP, all sampled from three sites near Mazatlán, Mexico. As a result, genetic diversity across the Central and Southern ETP remains largely uncharacterized. Moreover, GenBank currently lists only two accessions explicitly identified as *A. laticeps*, compared to more than 1,500 for *A. narinari* and over 140 for *A. ocellatus* [25–27]. Additionally, in a recent mitogenomic study of *A. ocellatus,* Marin [10] reported that three *A. laticeps* COI sequences from samples collected in Mexico and a single sequence collected in Peru did not present geographic genetic structure.

The most recent IUCN assessment (2019) classified the Eastern Pacific white-spotted eagle ray, *A. laticeps*, as Vulnerable, primarily due to the expansion of poorly regulated targeted and bycatch fisheries across its native range, as well as reports of declining landings in areas where the species was once common [28]. However, the assessment concluded that protected areas in the ETP, such as the Galápagos Marine Reserve, provided a buffer for this species [28]. In contrast, *A. narinari* and *A. ocellatus* are currently listed as Endangered, reflecting documented population declines of up to 80% and escalating human pressures throughout most of their distribution. Importantly, only *A. narinari* has species-specific information on actual exploitation levels, while the decline rates mentioned in the assessments for *A. ocellatus* and *A. laticeps* are inferred from broader taxonomic reconstructions of historical catch data [18,29,30]. Overall, comprehensive research on the ecology, population structure, and demographic trends of *A. laticeps* is needed to generate a robust assessment of its conservation status and to guide effective management actions across the Eastern Tropical Pacific. In this context, we present the first population genetic study of *A. laticeps*, based on 72 individuals collected from four countries spanning most of the species’ range.

## Methods

### Sampling

**Fig 1:**
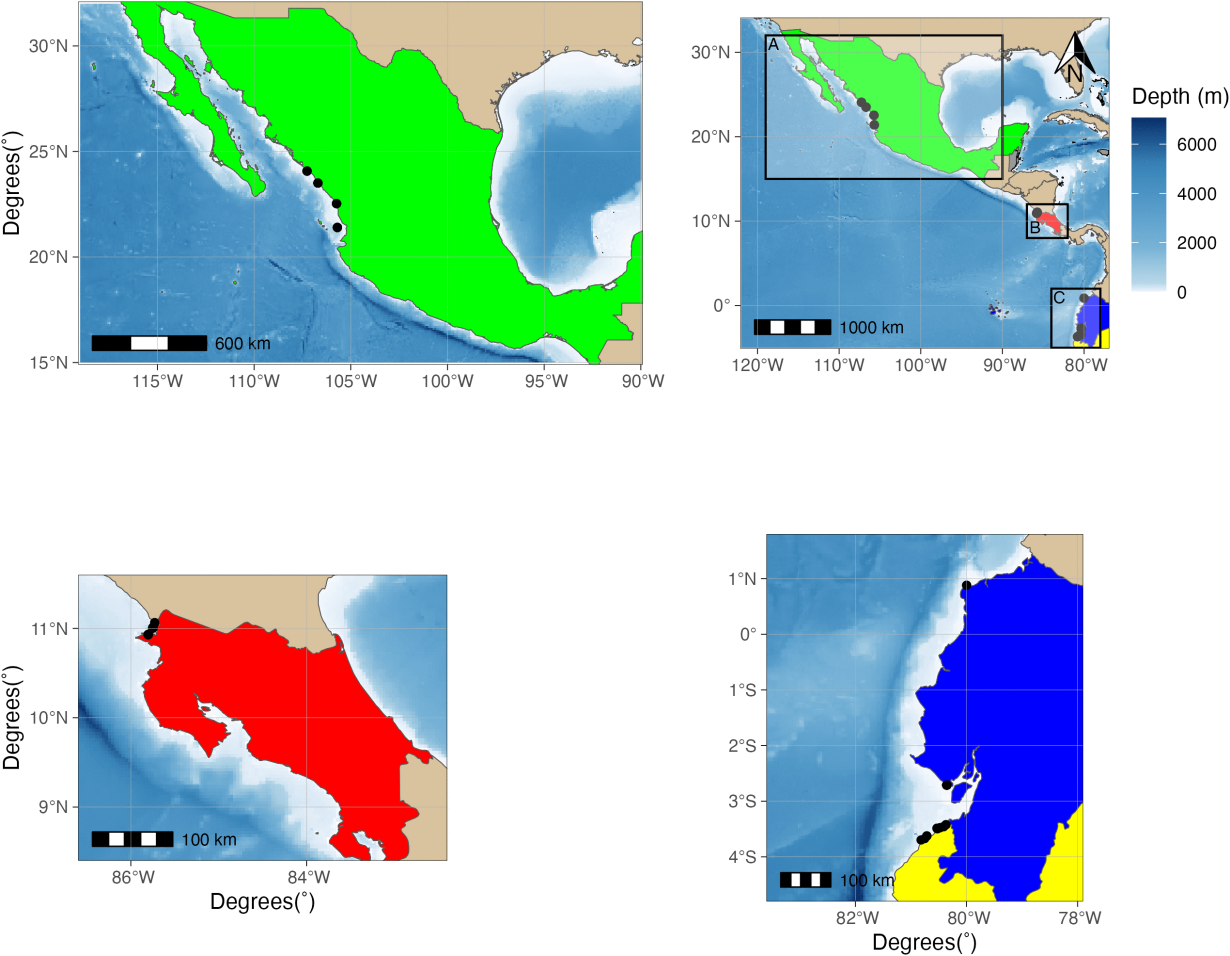
Study Area.

A total of 87 tissue samples were collected from four countries in the Eastern Tropical Pacific: 25 samples from the Gulf of California (Mexico), 20 samples from Ecuador, 22 from Peru and 20 samples from Costa Rica’s Pacific coast (Fig 1). Sample collection techniques varied among sites. Samples collected in Peru and Ecuador were obtained from fishermen as bycatch collected at multiple localities. Mexican samples were collected by scientific vessels researching shrimp trawling as detailed in [31]. Tissue samples collected in Costa Rica were taken from live individuals in the water using a Hawaiian sling. Regardless of the sampling technique, all tissue samples were preserved in 95% ethyl alcohol and stored at −20 ºC.

Fieldwork and sample collection were conducted in compliance with all relevant national regulations. In Mexico, samples were collected with the support of Proyecto Camarón del CRIAP Mazatlán, IMIPAS, and Ing. Darío Chávez Arrenquín, under collection permits PPF/DGOPA-238/13, PPF/DGOPA-052/14, and PPF/DGOPA- 002/16 issued by the Dirección General de Ordenamiento Pesquero y Acuícola (DGOPA). In Costa Rica, collections and export were conducted under permits R- SINAC-ACG-PI-026–2017 and R-CM-VERITAS-001–2024-OT-CONAGEBIO, issued by the Sistema Nacional de Áreas de Conservación (SINAC) and Comisión Nacional para Gestión de la Biodiversidad (CONAGEBIO). Samples from Perú were excluded from collection and export permits by the national authority, Ministerio de la Producción (PRODUCE). Samples from Ecuador were provided by local fishermen before the Ministerial Agreement protecting this species came into force. All sampling activities followed ethical and legal guidelines and were designed to minimize environmental impact.

This map displays the sampling sites of this study across the Eastern Tropical Pacific Ocean. Sampling was carried out in four countries highlighted in the panels A, B and C. (A for Mexico, B for Costa Rica and C for Ecuador and Peru). All country polygons used in this map were obtained from Natural Earth Data. Sampling sites are represented as black dots. The ocean floor is overlayed with a false-color representing bathymetry, ranging from white (0m) to dark blue (<-4000m) made with data from the General Bathymetric Chart of the Oceans (GEBCO) using the built-in bathymetry option from the R package ggOceanMaps [32,33].

### DNA extraction

DNA extractions were carried out using QIAGEN´s DNeasy ™ Blood and Tissue ® extraction kit (Qiagen, Germantown, MD, USA) following the kit’s handbook [34]. After extraction, DNA concentration and quality indexes were assessed using a NanoDrop™ 2000 spectrophotometer (Thermo Fisher Scientific, MA, USA). To check for genomic integrity, 3 μL of each DNA sample was mixed with 3 μL of BlueJuice™ loading buffer (10x). The loading buffer-sample mixtures were run on a 1% agarose gel with 1.5% SYBR™ Safe stain at 100V for 30 min.

### Primer design

The primer design followed a mixed marker approach (MMA), incorporating both nuclear and mitochondrial regions to adequately infer gene flow in a species that might exhibit male-biased dispersal [6,35]. The genetic regions analyzed (COI, CYTB and ITS2) were selected due to their use in previous eagle ray population genetics studies [6,7]. The forward CYTB primer, ANAR_CBF1, and the reverse ITS2 primer, Bat28Sr1 that we used were taken from Richards et al. [6]. All other primer sequences were designed in-house using Primer 3, with Chondrichthyes-wide sequence concatenations obtained from GenBank queries [36–38] (Table 1).

**Table 1:** List of primers used for this study.

Marker	Direction	Primer	Primer sequence	Amplicon length (bp)	Source
COI	Forward	CO1_Mylio_F2	5’-GcTTYATYGTcTGAGCCCA-3’	766	Homebuilt^a^
	Reverse	CO1_Mylio_R2	5’-AGYGGTTATGTGgTTGGCTTGA-3’		Homebuilt
CYTB	Forward	ANAR CBF1	5’-GAGGGGCAACTGTCATCACTAACC-3’	596	Richards et al, [6].
	Reverse	ANAR CRB2	5’-AGCAATTTGTCCGATGGTGA-3’		Homebuilt
ITS2	Forward	Bat5.8SmF1	5’-GCTACGCCTGTCTGAGGGTCGC-3’	740	Homebuilt
	Reverse	Bat28SR1	5’-ACAGGCTAGGCCTCGATCAGAAGG-3’		Richards et al, [6].

^a^Homebuilt: Primers designed for this study in Primer 3 using Chondrichthyes-wide sequence concatenations obtained from GenBank queries. ITS2.

### PCR amplification and sequencing

For the amplification of the COI and ITS2 regions, a total PCR volume of 30 μL was used, which comprised 3 μL of genomic DNA diluted to 20 ng/μL according to NanoDrop quantification, 19.3 μL of water, 3 μL of 10X Buffer, 0.9 μL of 50 mM MgCl_2_, 0.6 μL of 10 mM dNTPs, 1.5 μL of 10 μM forward and reverse primers, and 0.2 μL of 5 units/μL Taq Platinum Invitrogen. Samples were amplified using a ThermoFisher Scientific MiniAmp™ cycler. The thermocycler’s temperature profile was the same for both ITS2 and COI primers, except for the annealing step. Thermal cycling began with an initial denaturation at 94 ºC for 5 min, followed by 35 cycles of denaturation at 94 ºC for 30 s, annealing at 61 ºC (63 ºC for the ITS2 region) for 30 s, and extension at 72 ºC for 45 s. A final extension was carried out at 72 ºC for 5 min. For ITS2 samples that didn´t amplify under these conditions, 0.3 μL of BSA (1 mg/ml) were incorporated into the PCR mix, adjusting the water volume to maintain a total final volume of 30 μL.

For the amplification of the CYTB region, a total PCR volume of 27 μL was used, which included 3 μL of genomic DNA diluted to 20 ng/μL according to NanoDrop™ quantification, 14.35 μL of water, 2.7 μL of 10X Buffer, 0.81 μL of 50 mM MgCl_2_, 0.54 μL of 10 mM dNTPs, 2.7 μL of 10 μM for both primers and 0.2 μL of 5 units/μL of *Taq* Platinum Invitrogen. Amplification was carried out using a ThermoFisher Scientific MiniAmp™ cycler. The temperature profile started with an initial denaturation at 94 ºC for 5 min, followed by 35 cycles of denaturation at 94 ºC for 15 s, annealing at 60 ºC for 30 s, and extension at 72 ºC for 1 min. A final extension was performed at 72 ºC for 7 min.

All PCR reactions included a sample-free negative control to check for reagent contamination. Following thermal cycling, samples were run on a 1% agarose gel with 1.5% SYBR™ Safe stain, alongside a 100 bp ladder, at 100 V for 30 min to check for amplicon size, specificity, and negative control contamination. Amplicons were sequenced by Sanger capillary electrophoresis using an ABI 3730xl DNA Analyzer (Applied Biosystems). Both forward and reverse strands were sequenced for all samples. Chromatograms were visually inspected, low-quality regions were trimmed, and reads were assembled into consensus sequences. Samples that resulted in ambiguous results were purified and re-sequenced until it was possible to assemble a consensus sequence from forward and reverse reads.

### Base calling and sequence alignment

Electropherograms were trimmed and checked for base-specificity before assembling forward and reverse reads into a consensus sequence using the De Novo Assemble tool from Geneious ®. Consensus sequences were visually inspected against electropherograms in Geneious ® to resolve ambiguous base calls. Any base calls that could not be visually resolved retained their IUPAC uncertainty codes (e.g.,. R, S, N, etc.). After base-calling, sequences were aligned using Geneious´ ClustalW tool, with one alignment performed per region (CYTB, COI, ITS2). The alignments were trimmed to ensure all sequences had the same length within each marker. The COI and CYTB alignments were then concatenated into a separate 1155 bp alignment to capture phylogenetic groupings between loci. Sequence alignments were stored as FASTA files.

### Data processing and analysis

#### Haplotype networks.

Sequence alignments were analyzed using the R packages pegas, adegenet, ade4, apex, mmod, poppr, and msa within the RStudio environment [39–47]. Alignments were imported into R as FASTA files and then converted into a dna.bin type object to enhance analysis efficiency and reduce memory usage. Haplotypes were extracted from the concatenated COI and CYTB alignment using the pegas function haplotypes(). TCS Haplotype networks were inferred using the haplonet() function from pegas. The degree of relatedness between haplotypes in the network was expressed as Hamming distances (the pairwise distance between strings of equal length expressed as the number of positions in which aligned characters differ) because some sequences had unambiguous base calls [48]. The relationships between haplotypes were further illustrated in a heatmap of the Hamming distances between haplotypes visualized using the gplots package [49,50].

#### Fixation indexes, AMOVA and treestructure analyses.

To estimate pairwise and global-level fixation indexes, the FASTA alignment files of each marker, as well as the concatenated COI and CYTB marker, were transformed into *genind* objects using the multidna2genind() function [45]. This type of object was selected because it allows for flexible handling of hierarchical grouping structures. Sequences in each alignment were grouped into strata according to their country of origin, which we used as a hypothesis for defining and testing population structure.

Global fixation indexes including GST, Phi_st, Jost’s D, and diversity measurements were calculated from all markers, including the concatenated COI and CYTB marker. *Hs* and *Ht* were only calculated for the nuclear ITS2 marker. All global fixation and diversity statistics were estimated using the diff_stats() function from the mmods package [46]. Pairwise fixation indexes were only estimated from the concatenated mitochondrial markers. The pairwise fixation indexes we calculated included Nei’s GST, Hedrick’s GST and Jost’s D, which were selected because they represent distinct concepts, and respond differently to genetic diversity within populations [50]. Jost’s D calculates differentiation directly, providing a monotonic and accurate representation of subpopulation relatedness, which does not vary with intra-population genetic diversity *Hs* [51]. In contrast, early versions of GST, such as Nei’s GST, fail, to indicate differentiation in populations when genetic variability within populations is high. Therefore, even if populations share no alleles, Nei’s GST may suggest no differentiation when *Hs* is high [50,51]. Despite its limitations, Nei’s GST was included in this study because it was used in previous genetic delimitation studies of the *Aetobatus* genus and, therefore, it allowed us to make direct comparisons of population structuring with other eagle ray species [6,7]. Confidence intervals for global differentiation indexes, such as Nei’s Gst, Hedrick’s Gst, and Jost’s D, were calculated using the summarise_bootstrap() function from the mmod package [46]. To determine whether genetic variation was greater between or within populations, the popper.amova() function from popper was used [41]. To test if the differences between within and between group variation of the concatenated marker were significant a 999 permutations Monte Carlo test was performed using adegenet’s randtest() function [40].

TreeStructure (TS) analysis was used to infer the number of independently evolving populations among the analyzed sequences and to evaluate whether the data supported the geographic groupings proposed for the AMOVA and fixation index analyses [52]. The TS algorithm detects populations in time-scaled phylogenies by grouping subsets of tips (individuals) that present distinct coalescent patterns [52]. To assess whether a subset of tips descending from a given node represents a single population, TreeStructure applies a non-parametric test that compares the rank order of nodes within the subset against a Kingman coalescent null model. Significant deviations indicate the presence of distinct populations at that node [52]. Coalescent patterns were used to delimit populations, as they reflect shared evolutionary histories [53,54]. Recent coalescent events are consistent with demographic bottlenecks, whereas deeper and more dispersed node aggregations are indicative of population expansions [53,54].

#### Divergence time estimation.

We inferred Bayesian phylogenies using BEAST2 for all the markers (COI, CYTB, and ITS2) independently (S1–S3 Figs), as well as the for the concatenated COI and CYTB dataset. Population and divergence time analyses were conducted using the concatenated COI and CYTB alignment to maximize phylogenetic resolution through an increased number of informative sites. The ITS2 marker was excluded from intraspecific inference because no sequence variation was detected.

The concatenated mitochondrial phylogeny was rooted and time-calibrated using two previously estimated divergence events within the ANSC: (1) the split between the New World clades (*A. narinari* and *A. laticeps*) and the Indo-Pacific clade (*A. ocellatus*) at 3.7 Mya [6,7], and (2) the divergence between the ANSC common ancestor and the Western Pacific clades (*A. flagellum* and *A. narutobiei*) at 18.7 Mya [7]. Calibration nodes were implemented using NCBI accessions NC_022837 (for the *A. narutobiei*–*A. flagellum* node) and JN184054 (for the *A. ocellatus–A. narinari* node) [55,56].

To contextualize our samples within broader *A. narinari* diversity, we additionally included accession KX151649 (Caribbean) and accession BK072016 (Western Pacific) [10,57]. Although BK072016 is listed as *A. narinari* in GenBank, both the original authors and our phylogenetic analyses indicate that it corresponds to *A. ocellatus*. The MCMC run was set with 10 million iterations and a 10% Burn-in. The accession numbers of outgroup sequences used to construct the individual COI, CYTB, and ITS2 phylogenies are listed in S1 Table.

The concatenated COI and CYTB alignment, including outgroup sequences, was imported into BEAUTi2 [58], where it was partitioned by gene (COI and CYTB). Because both markers are mitochondrial genes located on the same strand and are expected to evolve under similar selective constraints, their clock and substitution models were linked. Substitution model uncertainty was accommodated using Bayesian model averaging implemented in the bModelTest package within BEAST2 [59]. The model space included substitution models parameterized by up to six relative substitution rates, with optional gamma‑distributed rate heterogeneity among sites, a proportion of invariant sites, and estimated (unequal) base frequencies. All substitution model parameters were jointly estimated during the MCMC. The MCMC run was set with 10 million iterations and a 10% Burn-in. A Yule-calibrated speciation model prior was selected, considering that all species analyzed, including the outgroups, are currently extant and represent distinctly evolving lineages. Birth rates were modelled as an uninformative prior with a gamma distribution, with the alpha parameter set to 0.01 and beta to 1000 to allow flexible estimation of speciation rates [60].

Tree posterior distribution densities and substitution model parameters were assessed in Tracer and in the BmodelAnalyser BEAUTi app [61]. bModelTest could not identify any single model with overwhelming (>50%) posterior support. The three substitution models with the highest posterior support were 121343 (23.9%), TN93 (11.75%), and 12334 (9%). Complete model-averaging results for the concatenated COI–CYTB dataset, as well as for each individual marker, are provided in the Supplementary Materials (S4–S7 Figs).

The best-supported tree was selected using TreeAnnotator, with a Burn-in percentage of 10%, a posterior probability limit of 0, and the target tree type set to maximum clade credibility. The node heights were rescaled to the posterior mean ages of each clade. The annotated phylogenies were visualized using the ggtree package in R [62].

#### Isolation modelling.

To understand the role of the environmental factors contributing to the geographic isolation observed among haplotypes, we compared an isolation-by-distance model and an isolation-by-resistance model, using depth as the primary explanatory variable. Depth was validated as a significant factor in species distribution using maximum entropy modelling in R, using eagle ray observations from the Eastern Tropical Pacific available on GBIF using the dismo package [63]. Our goal was to determine whether population differentiation was solely due to geographic distance or if environmental features provided a better explanation for the observed genetic diversity patterns. To achieve this, two geographic distance matrices were generated between sampling locations. First, a bathymetric map was obtained using the getNOAA.bathy() function from the marmap R package [64]. From this map, two transition probability matrices were created: one with a 300-depth limit and another with no depth limitation. From each transition matrix, we generated a geographic distance matrix between sampling points using a least cost paths algorithm via marmap’s lc.dist() function [64]. Then, using the gl.idb() function from the dartR package, we evaluated the relationship between each of these matrices and a matrix of pairwise genetic distances between samples collected from different localities, expressed as the linearized Jost’s D, with mantel tests [65].

## Results

### Population genetics

#### Database characterization.

Genomic DNA was extracted from 87 tissue samples. PCR amplification and Sanger sequencing generated 75 ITS2 sequences (471 bp), 81 COI sequences (649 bp), and 79 CYTB sequences (506 bp). High-quality sequences for all three markers were obtained for 73 samples. A further sample was excluded due to a frameshift-inducing indel in the COI sequence that resulted in a premature stop codon. All subsequent analyses were performed on the remaining 72 of the original 87 samples we collected.

#### Haplotype networks.

**Fig 2:**
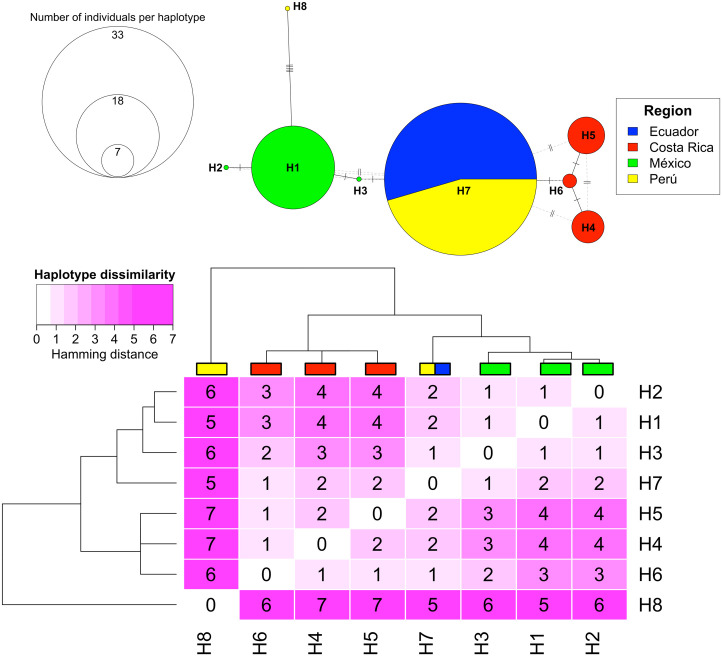
Haplotype Network and Hamming Distance Heatmap of concatenated COI and CYTB mitochondrial sequences. (A) depicts Haplotype Networks, where countries are represented by colors (Mexico = green, Peru = yellow, Ecuador = blue, and Costa Rica = red), and the diameter of the circles is proportional to the number of individuals that share each Haplotype. (B) Represents the relationships between haplotypes in a Hamming distance Heatmap. The heatmap is colored according to the number of nucleotides between each Haplotype. The color scales from white (no differences) to different scales of magenta (that darken with the number of nucleotide differences between sequences).

We identified eight haplotypes within the concatenated COI and CYTB regions from samples collected in the Eastern Tropical Pacific. However, we found only one ITS2 sequence type among all the samples collected. Fig 2 shows a clear geographic segregation of the concatenated COI and CYTB haplotypes. We identified three haplotypes from the samples taken in Costa Rica, three haplotypes from the Mexican samples, a unique haplotype from Peru and a shared haplotype between Ecuador and Peru. Most of the haplotypes we identified presented spatial autocorrelation, that is, the Hamming distances between haplotypes from the same region were smaller than the Hamming distance between haplotypes of different regions. Fig 2 shows the Hamming distances between haplotypes varied from one to seven nucleotides. The most divergent haplotype, H8, represented by a single sequence from Peru, differed from H1 (Mexico) by five nucleotides. Additionally, we observed a marked difference in internal diversity among populations. Only two haplotypes were detected among samples from Peru and Ecuador, whereas three haplotypes were identified in Mexico and three in Costa Rica. Furthermore, individuals were more evenly distributed among haplotypes in Costa Rica, than Mexico, Peru or Ecuador. All DNA sequences generated in this study were submitted to the GenBank database under accessions PX243675 to PX243747 for the CYTB gene, PX339970 to PX340042 for the ITS marker, and PX289121 to PX289193 for the COI gene.

#### Heterozygosity, haplotype and nucleotide diversity.

Subpopulation heterozygosity found with the ITS2 marker (*Hs*) 0.24428731 was almost two times smaller than total heterozygosity (*H*t) 0.5185224. When analyzing the concatenated COI-CYTB alignment we found that, on average, there were approximately 1.013831e-03 base differences per site between any two sequences, with a variance of 5.353463e-07 bases. Globally, haplotype diversity (*Hd*) was notably high, at 72.1% with a variance of less than 0.5%, indicating a very high probability of finding two distinct haplotypes when randomly sampling pairs of sequences. When analyzed separately, both mitochondrial markers had similar levels of nucleotide diversity, but lower levels of haplotype diversity (Table 2).

**Table 2:** Estimates of heterozygosity, nucleotide and haplotype diversity for each marker and the COI-CYTB concatenated dataset. Values for total and subpopulation heterozygosity are only reported for the biallelic nuclear marker ITS2.

Locus	Subpopulation Heterozygosity (*Hs*)	Total Heterozygosity (*Ht*)	Nucleotide Diversity (*Nd*)	Haplotype (Sequence) Diversity (*Hd*)
**Concatenated COI-CYTB**			1.01E-03	0.72108067
**CYTB**			5.85E-04	0.651087309
**COI**			1.28E-02	0.683024691
**ITS2**	0.24428371	0.5185224	0	0

#### Analysis of molecular variance.

The analysis of molecular variance (AMOVA) of the concatenated sequences revealed that genetic variation was higher between populations (68.98%) than within populations (31.02%) (Table 3). These differences between populations were statistically significant (p = 0.01) as demonstrated through Monte Carlo test with 999 permutations. The observed value of the phi statistic was almost five times higher than the expected value under a model that assumes no genetic structure.

**Table 3:** Results from the Monte Carlo Test of the Phi statistic and the genetic variation components.

Monte Carlo Test			
Expected φ Value	Observed φ Value	P-value^a^	Variance
0.329629e-04	0.5970928	0.01	3.948013e-04
**Variation composition**			
**Variation Component**	**Sigma**	**Variation (%)**	
**Between Populations**	0.5970928	68.98161	
**Within Populations**	0.2684898	31.01839	
**Total**	0.8655826	100	

^a^The P-values estimated in the upper-half of Table 2 were computed by comparing the distribution of simulated Phi statistic (φ) values from a null model to the actual observed Phi Statistic (φ). The simulated null mod (φ) distribution was obtained through 999 iterations.

#### Fixation indexes.

For the mitochondrial markers all fixation indexes, both in global and in pairwise comparisons, were high and in some cases equal to 1 (Table 4). At the global level, Phi_st, the proportion of inter-population genetic variance, presented the highest level of differentiation. At the pairwise level, Hedrick’s standardized GST and Jost’s D showed identical levels of differentiation. Nei’s GST presented the lowest differentiation values both at the global and pairwise levels.

**Table 4:** Global and pairwise fixation indexes.

Global Comparisons				
	Fixation Measurments			
Locus^a^	Nei’s Gst	Hedrick’s_st	Jost’s D	Phi_st
**Concat Mt-marker**	0.631452	0.935982	0.7897348	0.942614
**CYTB**	0.7024831	0.9374472	0.7405182	0.9464214
**COI**	0.8487723	0.9764409	0.8001391	0.9773822
**ITS2**	0.528885	0.7932771	0.4838477	0.7616545
**Pairwise Subpopulation Comparisons (Concat Mt-marker)**				
	**Fixation Measurement**			
**Pairing**	**Nei’s GST**	**Hedrick’s GST**	**Jost’s D**	
**Ecuador-Costa Rica**	0.51442308	1	1	
**Ecuador-Mexico**	0.76573337	1	1	
**Ecuador-Peru**	0.01728844	0.03617325	0.03617325	
**Costa Rica-Mexico**	0.37678869	1	1	
**Costa Rica-Peru**	0.44761357	1	1	
**Mexico-Peru**	0.67621665	1	1	

^a^Global fixation indexes were calculated for all loci. Pairwise fixation indexes between subpopulations were only calculated for the concatenated COI and CYTB mitochondrial markers.

Table 4 presents the results for each locus and the concatenated sequences. When analyzed separately, both mitochondrial markers presented higher values for all fixation indexes, with COI sequences showing the highest differentiation. On the other hand, ITS2 sequences had a lower differentiation than the mitochondrial markers, both individually and when concatenated. However, except for Jost’s D, the fixation indexes were still above 0.5.

At the pairwise level, gene flow between most subpopulation pairs appeared to be substantially restricted. Only Peru and Ecuador presented very low pairwise fixation indexes (i.e., < 0.5) across all measurements. Jost’s pairwise D and Hedrick’s pairwise GST were equal to one for all pairings except for Ecuador and Peru. On the other hand, Nei’s Pairwise GST showed that no two subpopulations were completely differentiated. Interestingly, according to Nei’s pairwise GST, the subpopulations from Ecuador and Peru were more closely related to the Costa Rican subpopulation than to the Mexican subpopulation. Conversely, the Mexican subpopulation was more closely related to the Costa Rican population than to the other two subpopulations.

### Delimitation of population structure

**Fig 3:**
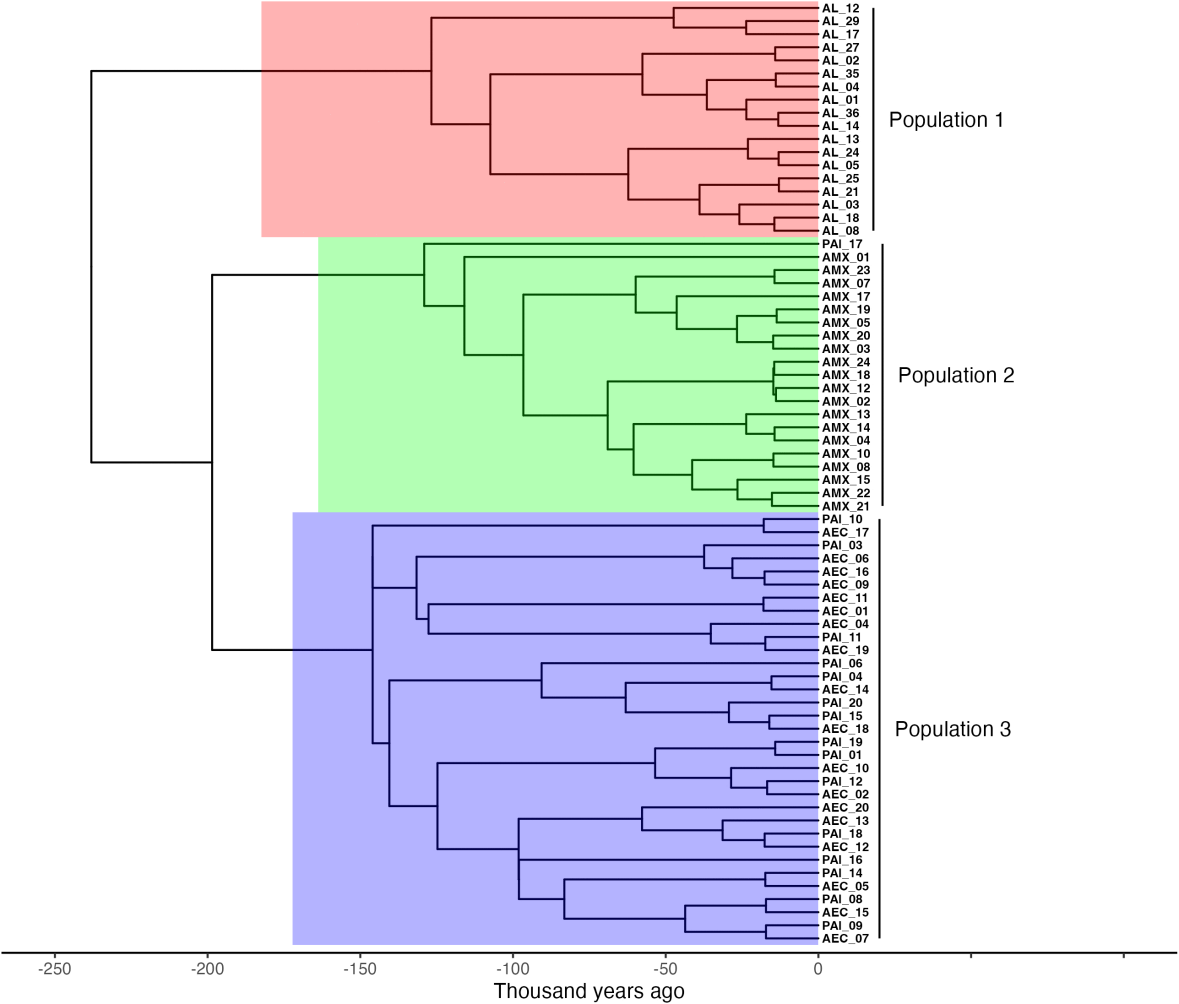
Analysis of coalescence patterns Treestructure. Population assignment of sequences resulting from Treestructure analysis of the time calibrated phylogeny of the concatenated COI-CYTB marker. Assigned populations are coded in color: (Population 1: red, Population 2: blue, and Population 3: green).

Treestructure analysis of the concatenated COI-CYTB dataset indicated the presence of three populations that closely correspond to the geographic origin of the samples (Fig 3). The first population included samples collected in Costa Rica exclusively. The second population encompassed sequences collected from both Ecuador and Peru, and the third population included all samples collected in Mexico plus an additional sample (Pal_17), collected in Peru.

### Bayesian phylogenetic trees

#### Concatenated sequences COI and CYTB.

**Fig 4:**
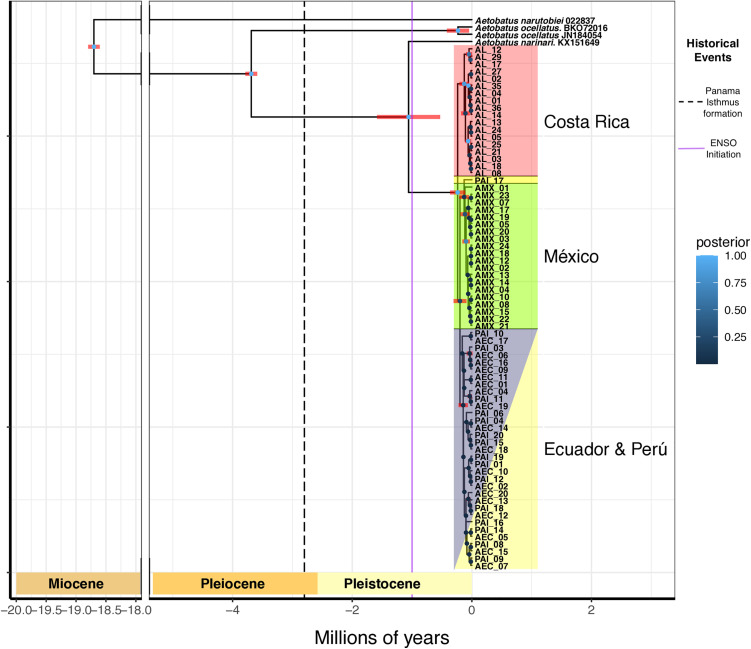
Calibrated Bayesian Phylogeny of Concatenated CYTB and COI sequences.

The Bayesian inference analysis resulted in a tree that shows that all the samples from our study fall into a single well-resolved node (posterior probability ~1) which diverged roughly 300 thousand years ago into three geographically isolated nodes. The first of these nodes only contained individuals from Costa Rica. The second node was composed of individuals from Mexico and one sample from Peru, Pal_17. The third node was composed of individuals from Ecuador and Peru (Fig 4). In most cases, relationships between individuals from the same populations were not properly resolved.

The divergence time between our sequences from the Eastern Tropical Pacific and *A. narinari* accession KX151649, sequenced from a specimen collected in the Florida Keys was estimated to be about 1.5 million years ago (Fig 4).

Most recent common ancestor calibrated Bayesian phylogeny of the concatenated COI and CYTB sequences. The dots at each node are colored according to their resolution’s posterior probability, lighter tones of blue signify a high posterior probability and a reliable resolution. The red horizontal bars over each node represent the 95% Highest Posterior Density on which each node could land on the temporal scale. The dotted vertical line represents the estimated timing for the formation of the Panama Isthmus according to O’Dea et al. [66]. The vertical lilac line represents the intensification of the temperature gradient of El Niño Southern Oscillation (ENSO) events according to Fedorov et al. [67]. The branches are underlaid with colored boxes indicating the country of origin of each individual.

### Distance vs resistance isolation modelling

**Fig 5:**
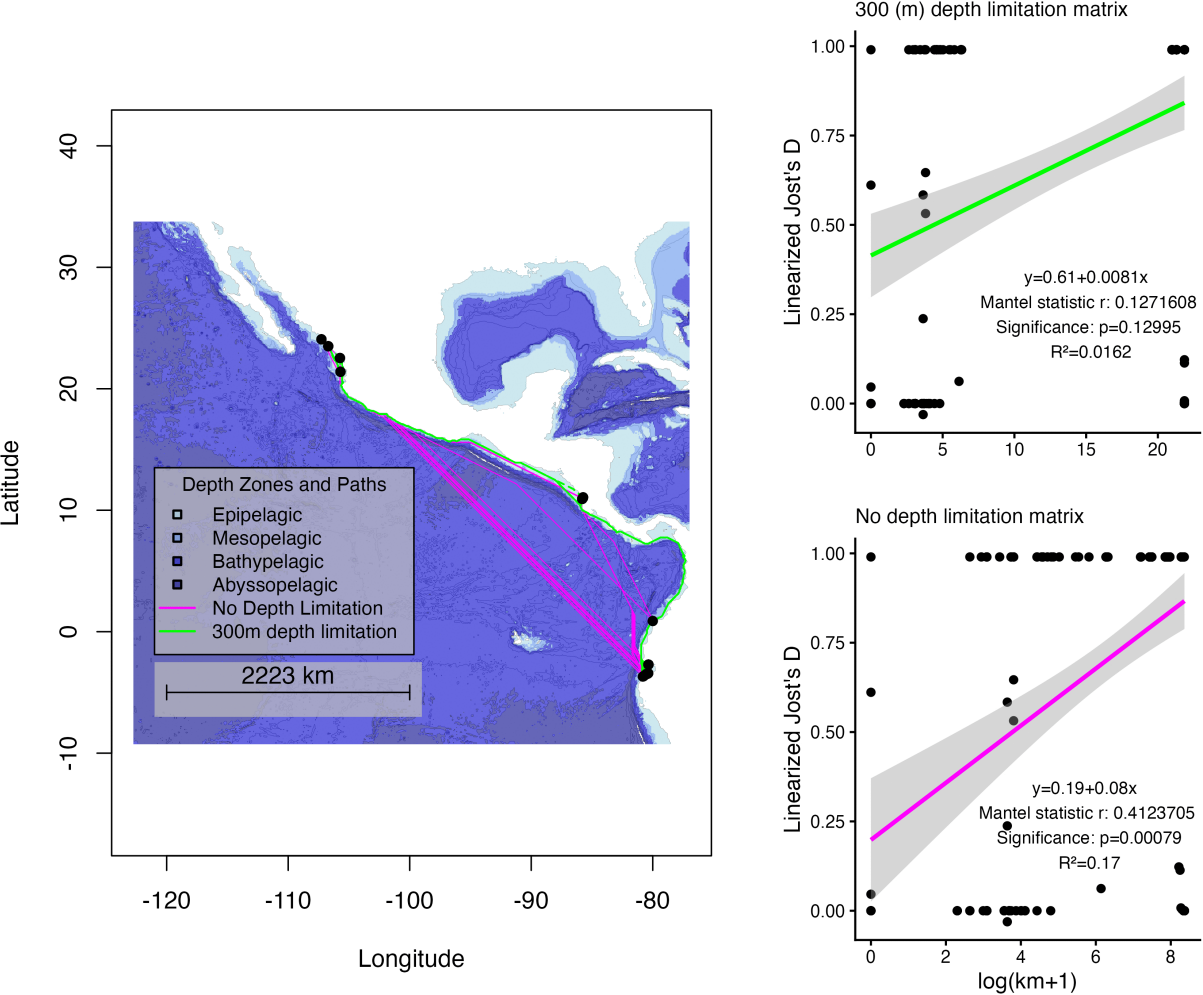
Least Cost Paths Map: 300-meter depth limitation vs. No depth limitation. (A) illustrates the study area and is colored according to depth zones. Darker colors represent deeper parts of the ocean. The magenta lines overlaid on the depth zones represent the shortest possible paths individuals could take irrespective of depth. The light green lines that border the coastline represent the shortest potential paths individuals would have to take when a 300-meter depth limitation was inputted into the model. Black dots represent sampling sites. (B) and (C) represent the regression between geographic distances between sampling sites and genetic distances between subpopulations under different depth constraints. Geographic distances were log-transformed to make the variation between the vertical axis and the horizontal axis similar in scale. This map was created using the marmap package using data from the NOAA’s ETOPO 2022 Arc-second Global Model [64,68].

We found that the matrix with no depth limitation explained more of the genetic variation and was more significant than the matrix with the depth limitation, however, the determination coefficients (R^2^) in both Mantel tests were lower than 0.5. (R^2.^ = 0.0162, and p = 0.125, and R^2^ = 0.17 and p = 0.00071, respectively) (Fig 5).

## Discussion

### Genetic diversity and connectivity of eagle rays in the Eastern Tropical Pacific

#### Mitochondrial diversity.

We found strong genetic structure accompanied by relatively low genetic diversity among eagle ray populations in the Eastern Tropical Pacific. Based on the concatenated mitochondrial markers COI and CYTB, we identified eight novel haplotypes showing clear geographic segregation. Of the eight haplotypes identified, only one (H7; Fig 2A) was shared between localities, occurring in both Ecuador and Peru; all others were geographically exclusive. Two haplotypes (H2 from Mexico and H8 from Peru) were represented by sequences containing ambiguous base calls. The representative sequence of H2 (AMX_02) displayed an R ambiguity at position 126 of the concatenated alignment, distinguishing it from other Mexican sequences. Haplotype H8 (PAL_17) contained four ambiguous sites; however, its clustering with Mexican haplotypes in the network was driven by a shared adenine at position 126 of the concatenated COI–CYTB alignment rather than by these ambiguities. Fixation indices and AMOVA supported significant population structure consistent with geographic separation. Pairwise fixation indices indicated, in some comparisons, an absence of mitochondrial gene flow between populations, with the exception of Ecuador and Peru (Table 4). Overall, the results support the presence of three genetically distinct populations: Mexico, Costa Rica, and a single panmictic unit comprising Ecuador and Peru. These patterns are consistent with philopatry and limited dispersal capacity [69–72].

While the haplotypes were mostly unique to their sampling locality, they exhibited limited divergence. Differing by up to seven base substitutions (with an average of 3.1622 base substitutions) in an 1155 bp alignment of concatenated CYTB and COI sequences. A similar trend was observed in high haplotype diversity (*Hd* ≈ 0.7) accompanied by low nucleotide diversity (*π* ≈ 0.004), suggesting recent demographic expansion from a smaller ancestral population, because rapid population growth typically results in the appearance of new genetic variants [73–75]. However, due to the short time scale, the number of mutations that can arise between these variants is limited [76–78]. These findings, together with the geographic distribution of haplotypes, suggest that reduced gene flow among populations is a relatively recent phenomenon and may reflect founder effects as a mechanism driving diversification within *Aetobatus* over short geo-temporal scales [79]. Although most haplotypes were unique to their sampling locality, they exhibited low levels of sequence divergence.

### Population structure

The combined evidence from haplotype networks, AMOVA, and coalescent analyses supports, based on our data, the presence of three populations in the Eastern Tropical Pacific: Mexico, Costa Rica, and a shared Ecuador–Peru population, each exhibiting different levels of internal diversity.

Subpopulation structure varied across the four sampling localities. Costa Rica had two haplotypes composed of a similar number of individuals, (H5 = 7 individuals, H6 = 8 individuals), and haplotype H7, comprising 3 individuals. Mexico and Ecuador/Peru displayed one predominant haplotype each, along with a few unique haplotypes represented by single individuals.

The subpopulation structure found in Costa Rica might be explained by how samples were collected in this locality. While samples collected from Ecuador, Peru and Mexico came mostly from by-catch, in Costa Rica tissue samples were collected from live individuals in shallow coastal areas near reefs and mangroves using a Hawaiian sling. Acoustic and satellite tagging studies on the Caribbean eagle ray, *A. narinari* have reported ontogenetic and sex-dependent niche-shifts [3,13,80]. In these studies, females and juveniles were found to spend longer periods in coastal areas, where food and protection are more easily accessible [13,80]. Furthermore, the same studies reported multiyear female site fidelity to coastal ecosystems. Given that in coastal shallow ecosystems, the probability of capturing females and pups is higher, and that only females exhibit a long-term site fidelity [3,13], collecting samples in shallow waters may result in more structured populations contrasted to deeper water sampling [3,13]. Overall, our findings reveal genetic structure among *A. laticeps* populations in the Eastern Tropical Pacific, similar to *A. narinari*, in the Western Atlantic [6,7].

### Nuclear diversity

Interestingly, while diversity in the mitochondrial markers was relatively high, variability in the nuclear marker was minimal, as evidenced by the single sequence type found with the ITS2 marker, and lower values in the fixation indices for this marker compared to the mitochondrial ones (Tables 1 and 3). The same asymmetry between mitochondrial and nuclear diversity has been observed in the other *Aetobatus* species. Both Richards et al. [6] and Sales et al. [7] reported fewer haplotypes with the ITS2 marker compared to COI and CYTB. For example, Richards et al. [6] identified only seven ITS2 haplotypes across three species sampled from the Eastern Pacific, Western Pacific, and central Atlantic, whereas the same individuals yielded 25 haplotypes using the CYTB marker alone. This asymmetry might be explained by the different mutation rates between the two mitochondrial coding sequences (COI and CYTB) and the nuclear non-coding ITS2, with mitochondrial markers mutating about 20 times faster on average in vertebrates [81]. This discrepancy could also reflect male-biased dispersal patterns, as mitochondrial genes are maternally inherited, and male-biased movement could lead to faster differentiation in mitochondrial markers than biparentally inherited markers [75]. Male-biased dispersal would be consistent with the reproductive and foraging behavior of eagle rays and aligns with patterns observed in other ovoviviparous elasmobranchs [13,36,82]. Nevertheless, additional nuclear markers are needed to better resolve population structure and gene flow dynamics in the region [83–85].

### Barriers to dispersal

Barriers to dispersal are relatively well understood for elasmobranchs. In a comprehensive literature review, Hirschfeld et al. [82] found that depth-related geographic features explain much of the genetic population structure observed across species with different habitat preferences. They showed that bathymetric features influence gene flow at varying spatial scales depending on ecological niche. For instance, benthopelagic species tended to differentiate across discontinuities in shallow habitats, whereas deep demersal species exhibited restricted gene flow across shallow barriers separating ocean basins, such as the Strait of Gibraltar. *A. laticeps*, like the other species from the *Aetobatus* genus, presents benthopelagic habitat preferences [3,13]. Depth may therefore impose a substantial constraint on gene flow between populations by forcing individuals to follow longer, more indirect coastal routes to move between locations [82]. Our findings further suggest that depth contributes significantly to the population structuring of eagle rays in the ETP. When comparing the depth limited (longer dispersal paths alongside the coastline) vs the no-depth limitation (shorter pelagic paths) scenarios, we found that the latter explained better the genetic variation between sampling sites (R^2^ = 0.0162, p = 0.125 and R^2^ = 0.17 and p = 0.00071 respectively) (Fig 5A, 5B, and 5C). However, in the absence of fine-scale discontinuities in suitable habitat, the role of depth as a barrier to gene flow in this species cannot be conclusively established. In addition, the Mantel tests yielded low correlation coefficients (Fig 5B, 5C), and the differences in significance between the isolation-by-distance (Fig 5A) and isolation-by-resistance (Fig 5B) models may reflect spatial autocorrelation in genetic variation that is not captured by the island model assumptions underlying the Mantel test [83–85]. Accordingly, further analyses using higher-resolution genomic data are needed to better resolve the processes shaping gene flow in the ETP [86].

As mentioned before, the population structure we found could be consistent with female philopatry, a behaviorally induced obstruction to gene flow, which is known to obscure and confound the effect that environmental barriers might have on population structure [82]. To better evaluate the role of bathymetry—and other environmental landscape features—in shaping population structure in this species, more controlled sampling designs are required. Future landscape genetic studies should implement balanced sampling schemes that incorporate interspersed populations and distance-controlled pairings and integrate data from a broader genomic framework [82].

#### Environmental influences on population structure.

The Bayesian phylogeny based on the concatenated mitochondrial markers (Fig 4) indicated an approximately 500,000-year divergence among the three genetic populations identified in the haplotype network, supporting our inference of recent divergence consistent with limited haplotype differentiation. The observed genetic structuring may be partially explained by climatic fluctuations during the mid-Pleistocene [1,3,13,87]. Over the last ten million years, climatic conditions in the Eastern Tropical Pacific have been highly unstable, with repeated significant sea level changes driven by glacial cycles [67,70,88–90]. During this period, sea surface temperatures experienced marked variability, characterized by recurrent warming phases [90]. Short-term oceanic variability fluctuated significantly, as evidenced by changes in the period and amplitude of El Niño Southern Oscillation (ENSO) temperature patterns [67]. Concurrently, oceanic current systems experienced major reorganization, including the re-emergence of the Walker circulation around three million years ago [91]. These environmental changes may help explain the genetic structure observed among populations [1,3,13,87]. The transition from a stable El Niño–like state to a cooler, more oscillatory regime around three million years ago could have fragmented a previously panmictic population by imposing thermal constraints and reducing suitable habitat availability [1,3,13,87]. Consistent with this, Osgood et al. [87] reported higher abundances of *A. laticeps* during strong El Niño years, when sea surface temperatures are elevated. Increased temperature variability across ENSO phases over the past ~1 million years may also have contributed to population declines, as extreme ENSO fluctuations have been associated with elevated mortality in multiple Eastern Tropical Pacific species [92–95]. In addition, glacially driven sea-level reductions likely diminished feeding and breeding habitats, potentially leading to further demographic contractions [96].

To test how paleoclimatic shifts impacted eagle ray population dynamics during the mid-Pleistocene, future studies could employ genome-wide analyses that estimate past demographic shifts and relate them to paleoclimatic events [97,98].

### Phylogenetics

#### Species delimitation and relationships with other species in the genus.

The Bayesian phylogeny based on the CYTB marker (S1 Fig) showed that our sequences clustered closely with accession MK340528, one of the reference specimens used by Sales et al. [7] to re-describe *A. laticeps* as a distinct species. This result supports the recognition of *A. laticeps* as a valid species occurring in the Eastern Tropical Pacific [5–8].

The concatenated COI-CYTB phylogenetic analysis indicated that our samples from the Eastern Tropical Pacific form a distinct and well-defined clade that separated from their Atlantic and Caribbean relatives approximately 1.8 million years ago. This clade was further split into three different branches around 500,000 years ago, displaying a geographically segregated pattern consistent with the structuring observed in the haplotype network. The divergence between eagle ray species in the Caribbean and Eastern Tropical Pacific appears to have occurred well after the final formation of the Panama Isthmus, estimated by O’Dea et al. [66] to have completed around 2.8 million years ago, roughly 1 million years before the split between the Caribbean *A. narinari* and our samples (Fig 4). However, the 95% HPD range of this divergence spans over 1 million years, meaning that the split could potentially have occurred up to 0.5 million years earlier. This temporal pattern is consistent with the conclusions of Sales et al. [7], who similarly reported that the divergence between Eastern Pacific and Caribbean lineages occurred after the widely accepted timing of Isthmus closure.

Our results add to the growing body of evidence indicating that the permeability of the Panama Isthmus to gene flow has varied over time, likely shaped by global climatic processes such as eustatic sea-level fluctuations and interglacial cycles [82,99–104]. At the same time, there is broad agreement that the biological separation of marine lineages between the Caribbean and the Pacific did not occur synchronously with the geological completion of the Isthmus [100]. Together, these observations underscore the need for more comprehensive, genome-wide studies of related taxa on both sides of the Isthmus to refine the timeline of seaway closure and its biological consequences.

### Conservation implications

The low genetic variability and limited gene flow observed among populations, together with the slow replenishment rates characteristic of eagle rays, suggest that the Eastern Tropical Pacific species *A. laticeps* may be more vulnerable to human impacts than previously recognized. As population sizes decline, their capacity to withstand environmental change is reduced.

Although *A. laticeps* is currently classified as Vulnerable by the IUCN, it faces threats similar to those affecting its sister species, in some cases potentially at greater intensity. For example, bycatch in shrimp trawl fisheries in the Gulf of Mexico is cited as a primary threat to *A. narinari* in the Caribbean. In contrast, the majority of shrimp landings in Mexico—both artisanal and industrial—occur along the Pacific coast, particularly in Sinaloa, Baja California, and Guerrero, where most of the country’s shrimping fleet operates [105] . Therefore, we suggest that Eastern Tropical Pacific eagle rays’ conservation status should be assessed far more conservatively. To this end, we recommend the following actions:

1 Support research aimed at identifying critical life-cycle habitats (e.g., nursery areas and feeding aggregations) essential for the long-term survival of eagle ray populations.
2 Improve systematic landing data collection across the Eastern Tropical Pacific to assess the impact of fisheries at both local and regional level.
3 Assess the demand for products derived from this species, both locally and internationally, to determine if it should be included in more comprehensive conservation frameworks like CITES.
4 Prioritize site-based conservation efforts assessed at the population level for this species in its preferred coastal habitats.
5 Explore further population structure using genomic data (e.g. SNPs) including new locations within the ETP.
6 Develop a formal petition to the IUCN to reassess the current conservation status of the species, at least in regions such as Mexico, based on the genetic isolation documented in this study and the combined impacts of fisheries pressure and habitat degradation.

## Conclusions

To date, this is the first study to assess the genetic diversity and structure of *Aetobatus laticeps* eagle rays in the Eastern Tropical Pacific, shedding light on a species that has been understudied. Our results confirm that spotted eagle rays in the continental stretches of the Eastern Tropical Pacific correspond to a single species, *Aetobatus laticeps*, and reveal the presence of three genetic groups (Mexico, Costa Rica, and Ecuador/Peru). This species’ populations presented low genetic diversity in the markers we tested. Nuclear and mitochondrial markers presented vastly different variation patterns. Mitochondrial markers (CYTB and COI) presented some genetic variability and a high level of geographic-genetic structure (S1 and S2 Figs). However, the nuclear marker ITS2 presented no variability and no discernible branching patterns (S3 Fig). Our data pointed to three possible mechanisms that could explain the population structure we encountered: a) Isolation by distance, suggested by the spatial autocorrelation of genetic distances, the branching patterns of the phylogenetic trees, and the relationship between haplotypes. b) Isolation by philopatry implied by the difference in genetic variability between nuclear and mitochondrial markers, and c) Isolation by resistance hinted at by the diet and habitat preferences of this evidenced by the higher correlation coefficient and lower significance level of the no-depth limitation Mantel test.

## Supporting information

S1 Table List of accessions used for Bayesian Phylogeny outgroups and calibration nodes for trees in the supplementary materials. (DOCX)

S1 Fig Bayesian Tree of CYTB sequences. S1 Fig is the most recent common ancestor time-calibrated Bayesian phylogeny of the CYTB marker. The dots at each node are colored according to their resolution’s posterior probability, lighter tones of blue signify a high posterior probability and a reliable resolution. The red horizontal bars over each node represent the 95% Highest Posterior Density on which each node could land in the temporal scale. The dotted vertical line represents the estimated timing for the formation of the Panama Isthmus, and the vertical lilac line represents the intensification of El Niño Southern Oscillation (ENSO) events.
(PDF)

S2 Fig Bayesian Tree of COI sequences. S2 Fig Illustrates a most recent common ancestor calibrated Bayesian phylogeny for sequences for the COI marker. The dots at each node are colored according to their resolution’s posterior probability, lighter tones of blue signify a high posterior probability and a reliable resolution. The red horizontal bars over each node represent the 95% Highest Posterior Density on which each node could land in the temporal scale. The dotted vertical line represents the estimated timing for the formation of the Panama Isthmus, and the vertical lilac line represents the intensification of El Niño Southern Oscillation (ENSO) events.
(PDF)

S3 Fig Bayesian Tree of (A) Time Calibrated ITS2 Sequences. S3 Fig is the most recent common ancestor time-calibrated Bayesian phylogeny of the ITS2 marker. The dots at each node are colored according to their resolution’s posterior probability, lighter tones of blue signify a high posterior probability and a reliable resolution. The red horizontal bars over each node represent the 95% Highest Posterior Density on which each node could land in the temporal scale. The dotted vertical line represents the estimated timing for the formation of the Panama Isthmus, and the vertical lilac line represents the intensification of El Niño Southern Oscillation (ENSO) events.
(PDF)

S4 Fig bModelTest output for the concatenated COI-CYTB phylogeny. S4 Fig represents the posterior support of each of the models included in the search space. The size of the models’ bubble is proportional to its posterior support. Model bubbles with a blue outline are inside the 95% HPD. Model bubbles with a red outline have at most 0.27% support.
(PDF)

S5 Fig bModelTest output for the COI phylogeny. S5 Fig represents the posterior support of each of the models included in the search space. The size of the models’ bubble is proportional to its posterior support. Model bubbles with a blue outline are inside the 95 % HPD. Model bubbles with a red outline have at most 0.27 % support.
(PDF)

S6 Fig bModelTest output for the CYTB phylogeny. S5 Fig represents the posterior support of each of the models included in the search space. The size of the models’ bubble is proportional to its posterior support. Model bubbles with a blue outline are inside the 95% HPD. Model bubbles with a red outline have at most 0.27% support.
(PDF)

S7 Fig bModelTest output for the ITS2 phylogeny. S5 Fig represents the posterior support of each of the models included in the search space. The size of the models’ bubble is proportional to its posterior support. Model bubbles with a blue outline are inside the 95% HPD. Model bubbles with a red outline have at most 0.27% support.
(PDF)
