# Supplementary material for: Phylogeography and population genetics of the white spotted eagle ray, Aetobatus laticeps Gill, 1865, in the Eastern Tropical Pacific
Source: PLoS One. 2026 May 18;21(5):e0349373. doi: 10.1371/journal.pone.0349373 (PMC13183237; doi:10.1371/journal.pone.0349373)
Supplement: S1 Table — (DOCX) [file pone.0349373.s001.docx]

**S1 Table. List of accessions used for Bayesian Phylogeny outgroups and calibration nodes**

| **Marker** | **Accession** | **Region** | **Species** | **Citation** |
| --- | --- | --- | --- | --- |
| CYTB | MK340528 | México | *A. narinari* | [7] |
| CYTB | MK340548 | Brazil | *A. narinari* | [7] |
| CYTB | FJ812188 | Hawaii | *A. narinari* | [6] |
| CYTB | BKO72016 | Philippines | *A.*sp | [10] |
| CYTB | JN184054 | N/A | *A. ocellatus* | [77] |
| CYTB | NC_022837 | N/A | *A. narutobiei* | [78] |
| ITS2 | FJ812206 | N/A | *A. flagellum* | [6] |
| ITS2 | FJ812181 | Hong Kong | *A. ocellatus* | [6] |
| ITS2 | MK340564 | Brazil | *A. narinari* | [7] |
| ITS2 | FJ812184 | México | *A. narinari* | [6] |
| ITS2 | FJ812183 | Florida | *A. narinari* | [6] |
| COI | NC_022837 | N/A | *A. narutobiei* | [78] |
| COI | JN184054 | N/A | *A. ocellatus* | [77] |
| COI | KX151649 | Florida | *A. narinari* | [79] |
| COI | BKO72016 | N/A | *A.* sp. | [10] |
