# Supplementary figures and images for: Phylogeography and population genetics of the white spotted eagle ray, Aetobatus laticeps Gill, 1865, in the Eastern Tropical Pacific
Source: PLoS One. 2026 May 18;21(5):e0349373. doi: 10.1371/journal.pone.0349373 (PMC13183237; doi:10.1371/journal.pone.0349373)

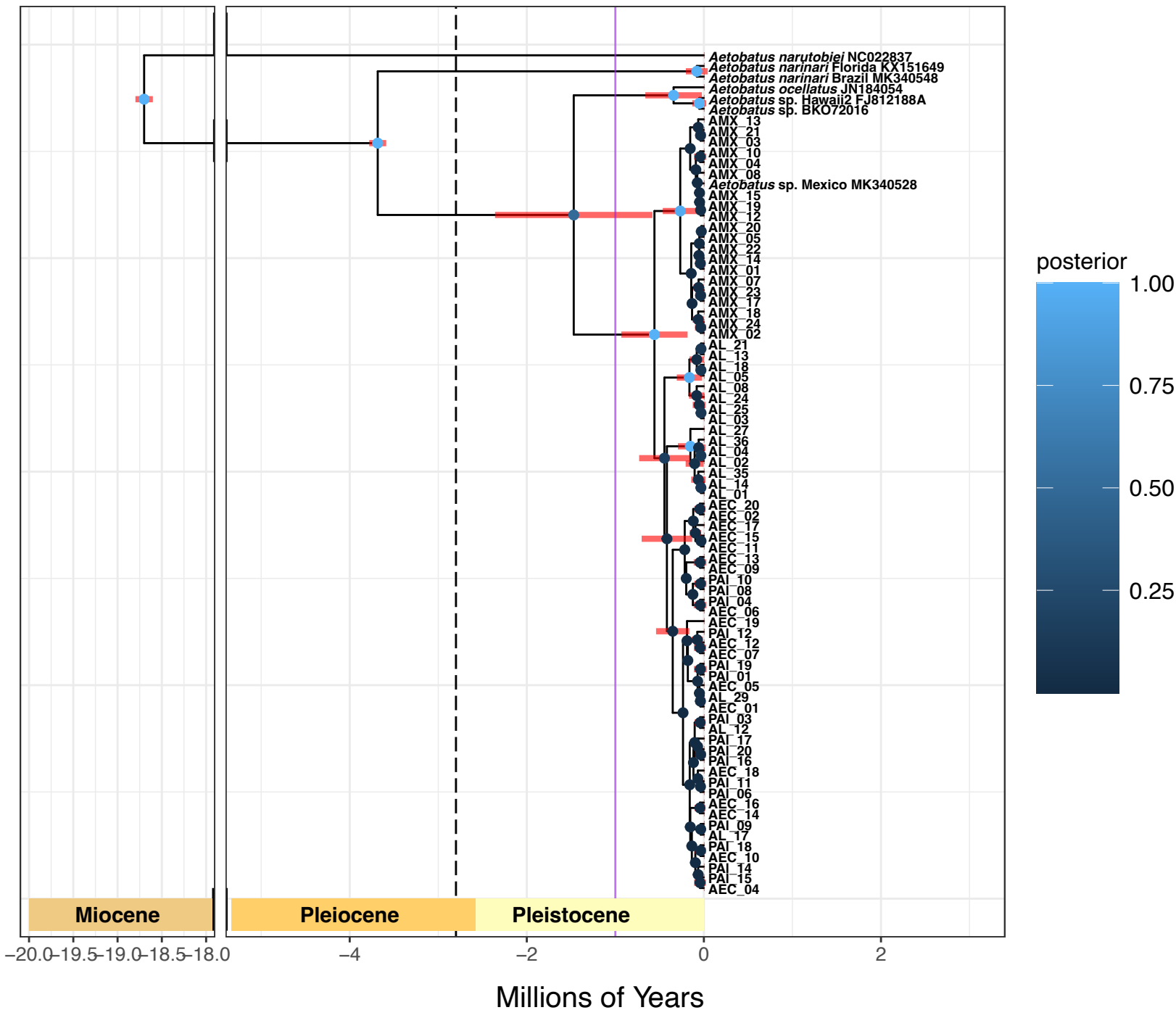

Supplement: S1 Fig — is the most recent common ancestor time-calibrated Bayesian phylogeny of the CYTB marker. The dots at each node are colored according to their resolution’s posterior probability, lighter tones of blue signify a high posterior probability and a reliable resolution. The red horizontal bars over each node represent the 95% Highest Posterior Density on which each node could land in the temporal scale. The dotted vertical line represents the estimated timing for the formation of the Panama Isthmus, and the vertical lilac line represents the intensification of El Niño Southern Oscillation (ENSO) events. (PDF) [file pone.0349373.s002.pdf]

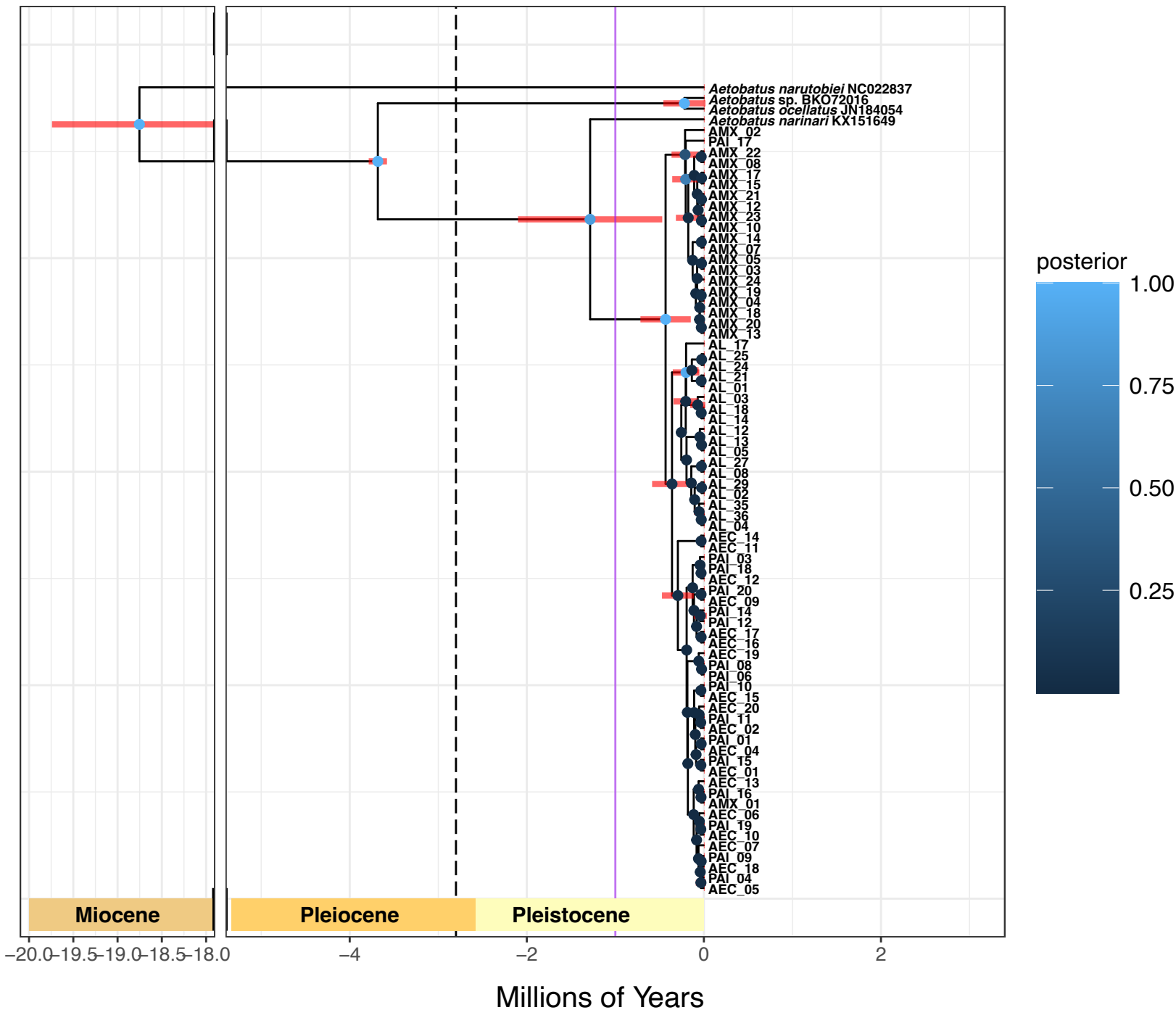

Supplement: S2 Fig — Illustrates a most recent common ancestor calibrated Bayesian phylogeny for sequences for the COI marker. The dots at each node are colored according to their resolution’s posterior probability, lighter tones of blue signify a high posterior probability and a reliable resolution. The red horizontal bars over each node represent the 95% Highest Posterior Density on which each node could land in the temporal scale. The dotted vertical line represents the estimated timing for the formation of the Panama Isthmus, and the vertical lilac line represents the intensification of El Niño Southern Oscillation (ENSO) events. (PDF) [file pone.0349373.s003.pdf]

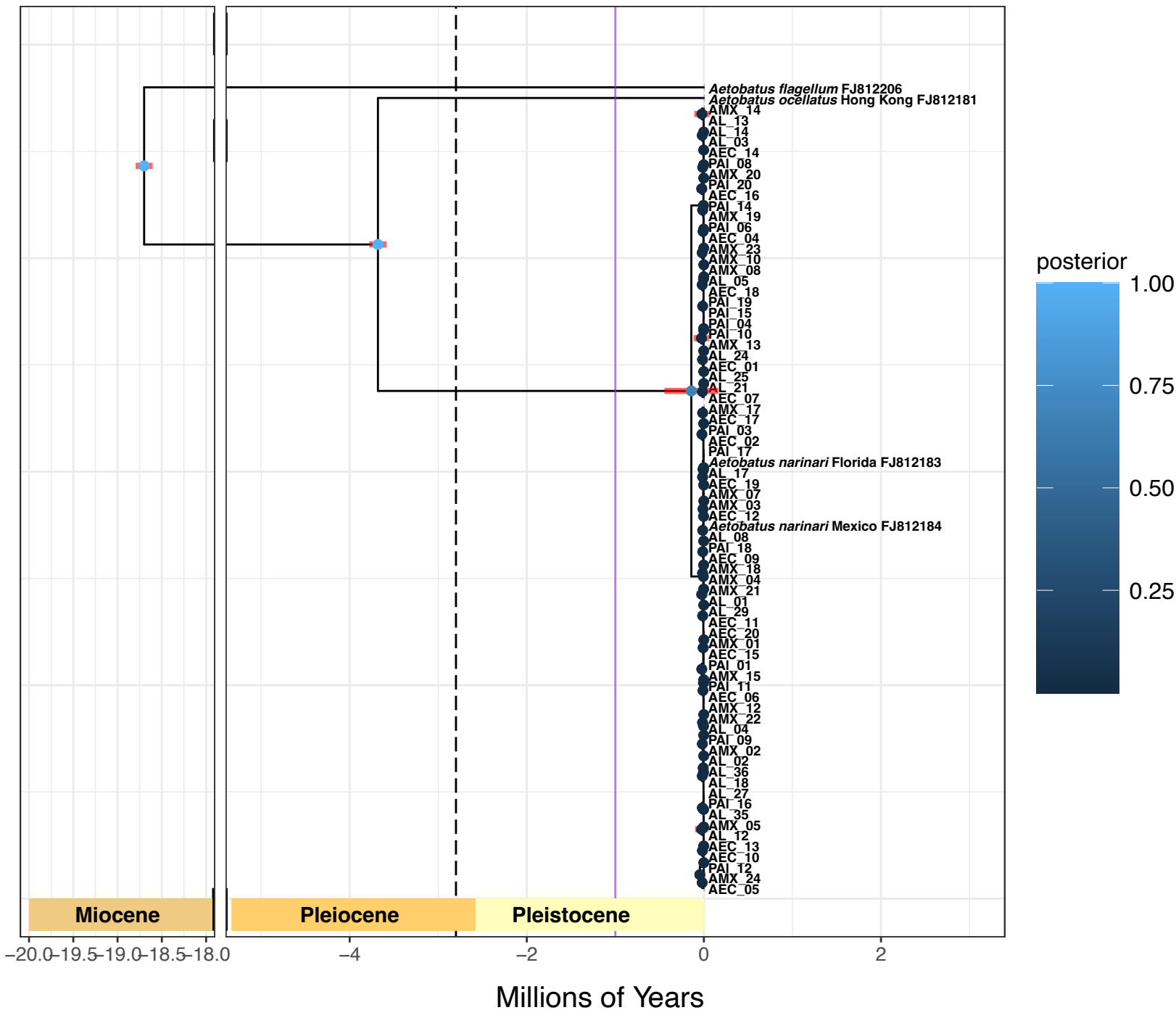

Supplement: S3 Fig — is the most recent common ancestor time-calibrated Bayesian phylogeny of the ITS2 marker. The dots at each node are colored according to their resolution’s posterior probability, lighter tones of blue signify a high posterior probability and a reliable resolution. The red horizontal bars over each node represent the 95% Highest Posterior Density on which each node could land in the temporal scale. The dotted vertical line represents the estimated timing for the formation of the Panama Isthmus, and the vertical lilac line represents the intensification of El Niño Southern Oscillation (ENSO) events. (PDF) [file pone.0349373.s004.pdf]
