# Supplementary material for: Phylogeography and population genetics of the white spotted eagle ray, Aetobatus laticeps Gill, 1865, in the Eastern Tropical Pacific
Source: PLoS One. 2026 May 18;21(5):e0349373. doi: 10.1371/journal.pone.0349373 (PMC13183237; doi:10.1371/journal.pone.0349373)

File: Corrected_unsplit_model.log item: substmodel

Models with blue circles are inside 95%HPD, red outside, and without circles have at most 0.27% support.

posterior support	cumulative support	model
23.49%	23.49%	121343
11.75%	35.23%	121131
9.28%	44.51%	123343
8.78%	53.29%	121134
8.14%	61.43%	123454
4.47%	65.89%	121341
4.37%	70.26%	121345
3.40%	73.67%	123345
3.32%	76.98%	123141
3.24%	80.22%	123143
3.24%	83.46%	123341
2.74%	86.20%	121323
2.45%	88.65%	123453
2.06%	90.71%	123145
1.58%	92.29%	123456
1.31%	93.60%	123451
1.26%	94.86%	123424
0.84%	95.70%	123323
0.75%	96.45%	121123
0.70%	97.16%	121121
0.56%	97.71%	121324
0.44%	98.16%	123324
0.27%	98.43%	121321
0.27%	98.70%	123423
0.26%	98.96%	123121
0.25%	99.21%	123124
0.24%	99.46%	123425
0.24%	99.70%	123321
0.16%	99.86%	123123
0.14%	100.00%	123421

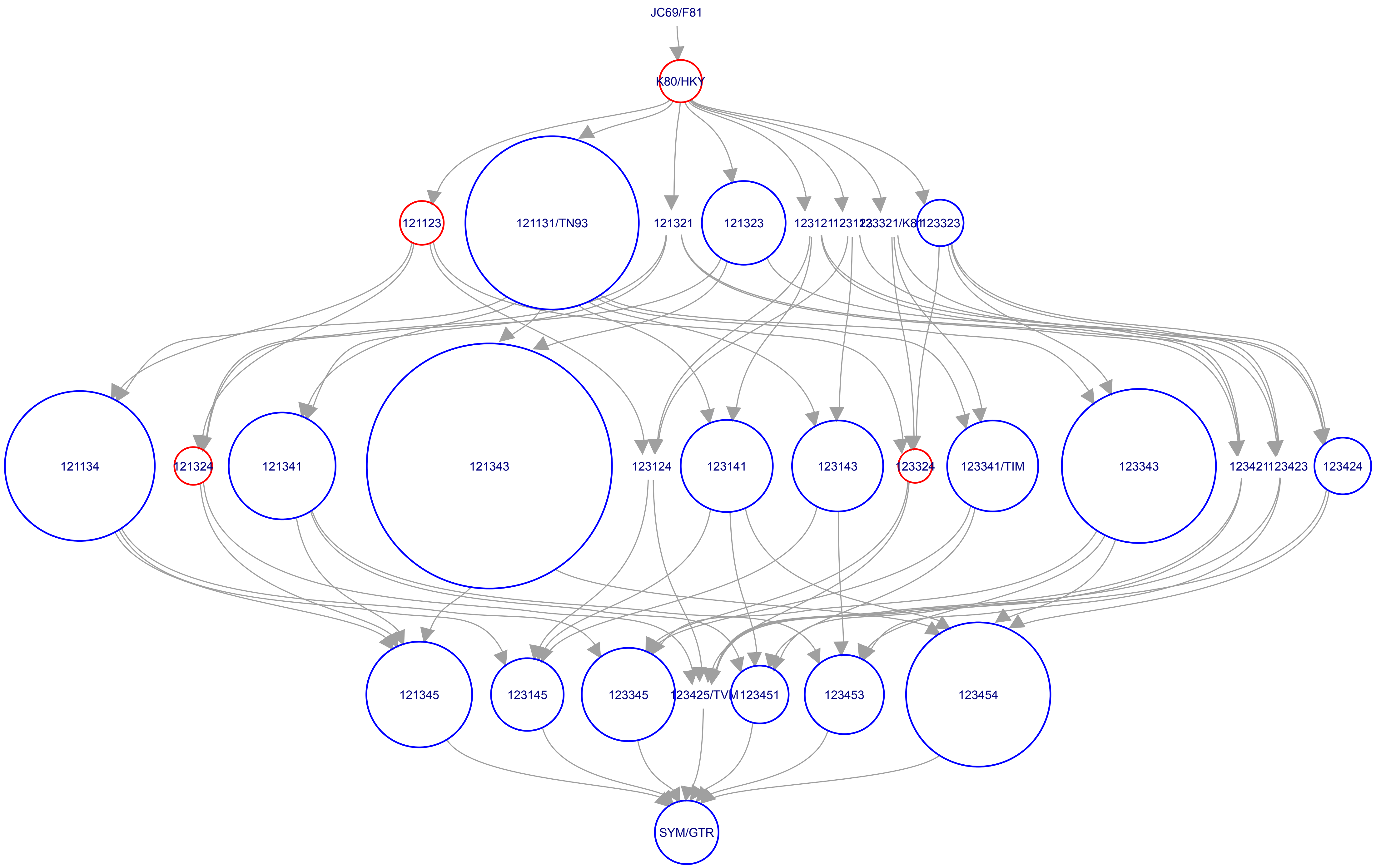

Supplement: S4 Fig — represents the posterior support of each of the models included in the search space. The size of the models’ bubble is proportional to its posterior support. Model bubbles with a blue outline are inside the 95% HPD. Model bubbles with a red outline have at most 0.27% support. (PDF) [file pone.0349373.s005.pdf]
