# Supplementary material for: Phylogeography and population genetics of the white spotted eagle ray, Aetobatus laticeps Gill, 1865, in the Eastern Tropical Pacific
Source: PLoS One. 2026 May 18;21(5):e0349373. doi: 10.1371/journal.pone.0349373 (PMC13183237; doi:10.1371/journal.pone.0349373)

File: Aetobatidae_2026_COI.log item: substmodel

Models with blue circles are inside 95%HPD, red outside, and without circles have at most 0.23% support.

posterior support	cumulative support	model
16.68%	16.68%	121121
11.03%	27.71%	121323
9.62%	37.33%	123121
7.29%	44.62%	121123
5.34%	49.96%	123321
4.93%	54.89%	121321
4.54%	59.44%	123323
4.12%	63.56%	123424
4.10%	67.66%	123123
3.57%	71.23%	121131
3.26%	74.48%	123124
2.70%	77.18%	121324
2.67%	79.85%	123421
2.56%	82.40%	121343
2.23%	84.64%	123324
2.16%	86.79%	123141
1.67%	88.46%	121134
1.44%	89.90%	123341
1.32%	91.22%	123423
1.09%	92.31%	123425
1.08%	93.39%	121341
1.03%	94.42%	123143
1.00%	95.42%	123343
0.92%	96.34%	123145
0.87%	97.21%	123454
0.76%	97.97%	123345
0.72%	98.69%	121345
0.61%	99.30%	123451
0.47%	99.77%	123453
0.23%	100.00%	123456

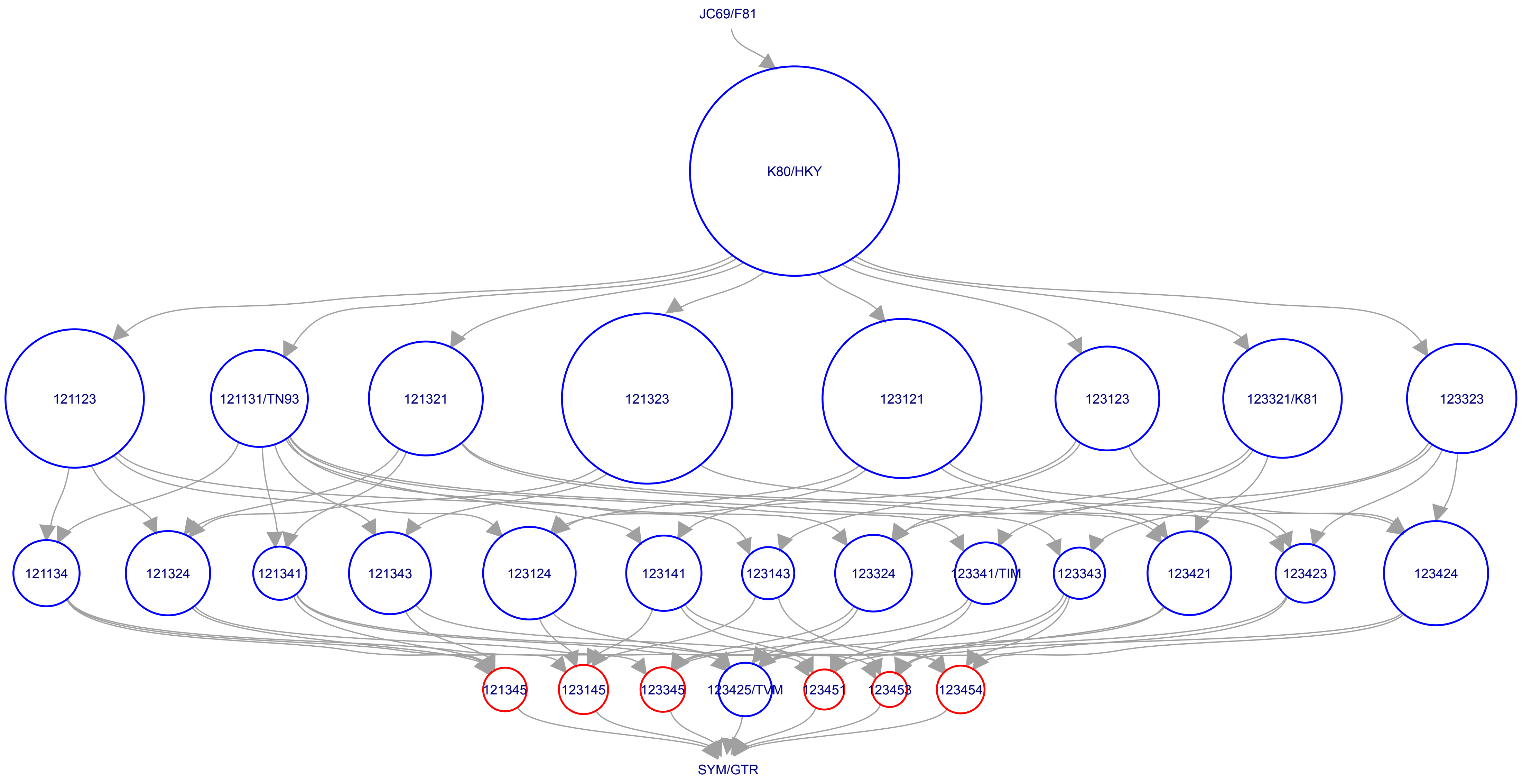

Supplement: S5 Fig — represents the posterior support of each of the models included in the search space. The size of the models’ bubble is proportional to its posterior support. Model bubbles with a blue outline are inside the 95 % HPD. Model bubbles with a red outline have at most 0.27 % support. (PDF) [file pone.0349373.s006.pdf]
