# Supplementary material for: Phylogeography and population genetics of the white spotted eagle ray, Aetobatus laticeps Gill, 1865, in the Eastern Tropical Pacific
Source: PLoS One. 2026 May 18;21(5):e0349373. doi: 10.1371/journal.pone.0349373 (PMC13183237; doi:10.1371/journal.pone.0349373)

File: Aetobatidae_2026-Cytb.log item: substmodel

Models with blue circles are inside 95%HPD, red outside, and without circles have 0.00% support.

posterior support	cumulative support	model
9.94%	9.94%	121323
8.74%	18.69%	123323
7.31%	26.00%	121123
7.27%	33.26%	123324
6.62%	39.88%	123424
5.65%	45.54%	121121
5.39%	50.93%	121324
4.48%	55.40%	123123
4.36%	59.76%	123425
4.13%	63.89%	123423
3.68%	67.57%	123124
2.94%	70.51%	121343
2.87%	73.38%	121321
2.78%	76.16%	123321
2.56%	78.71%	123343
2.53%	81.25%	123345
2.41%	83.66%	121134
1.89%	85.55%	123454
1.84%	87.39%	123121
1.63%	89.02%	121345
1.60%	90.62%	121131
1.51%	92.13%	123453
1.51%	93.65%	123421
1.48%	95.12%	123143
1.23%	96.36%	123145
1.08%	97.43%	123456
0.78%	98.21%	123341
0.74%	98.96%	121341
0.60%	99.56%	123141
0.44%	100.00%	123451

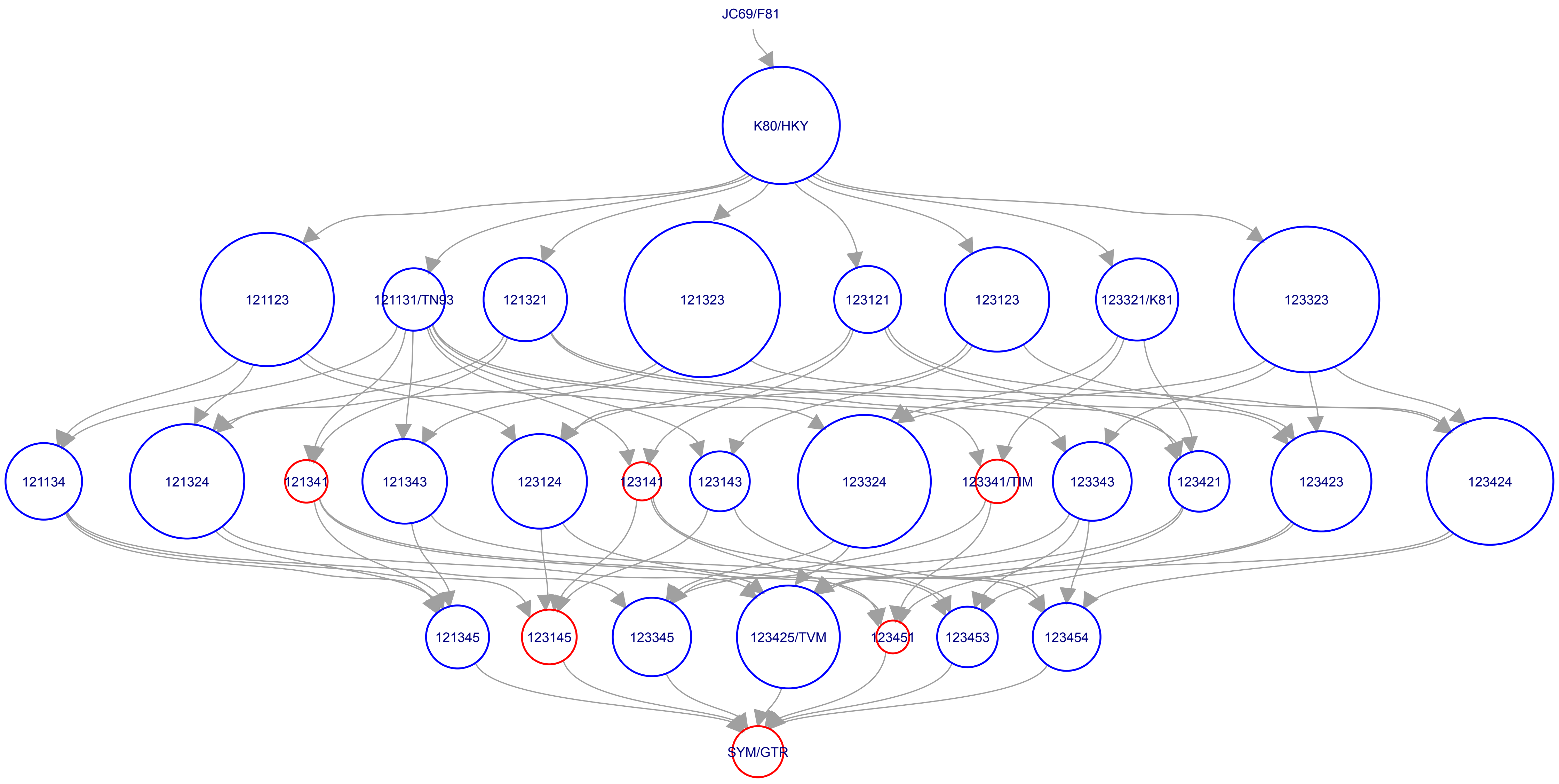

Supplement: S6 Fig — S5 Fig represents the posterior support of each of the models included in the search space. The size of the models’ bubble is proportional to its posterior support. Model bubbles with a blue outline are inside the 95% HPD. Model bubbles with a red outline have at most 0.27% support. (PDF) [file pone.0349373.s007.pdf]
