# Supplementary material for: Phylogeography and population genetics of the white spotted eagle ray, Aetobatus laticeps Gill, 1865, in the Eastern Tropical Pacific
Source: PLoS One. 2026 May 18;21(5):e0349373. doi: 10.1371/journal.pone.0349373 (PMC13183237; doi:10.1371/journal.pone.0349373)

File: Aetobatidae_2026_ITS2.log item: substmodel

Models with blue circles are inside 95%HPD, red outside, and without circles have 0.00% support.

posterior support	cumulative support	model
8.32%	8.32%	123343
7.14%	15.46%	123454
6.95%	22.42%	123453
5.79%	28.21%	123143
5.64%	33.85%	123345
5.53%	39.38%	123456
4.96%	44.34%	123145
4.60%	48.94%	123141
3.69%	52.63%	121131
3.64%	56.27%	123451
3.48%	59.75%	123341
3.10%	62.85%	123323
2.99%	65.84%	121134
2.87%	68.70%	121343
2.79%	71.49%	123423
2.66%	74.15%	123424
2.63%	76.78%	121341
2.40%	79.18%	111111
2.24%	81.42%	123425
2.23%	83.66%	121345
2.11%	85.77%	123123
2.06%	87.82%	123324
1.93%	89.76%	123124
1.88%	91.63%	123121
1.57%	93.20%	121121
1.44%	94.65%	121123
1.34%	95.99%	123421
1.19%	97.18%	123321
1.03%	98.21%	121324
0.93%	99.14%	121323
0.86%	100.00%	121321

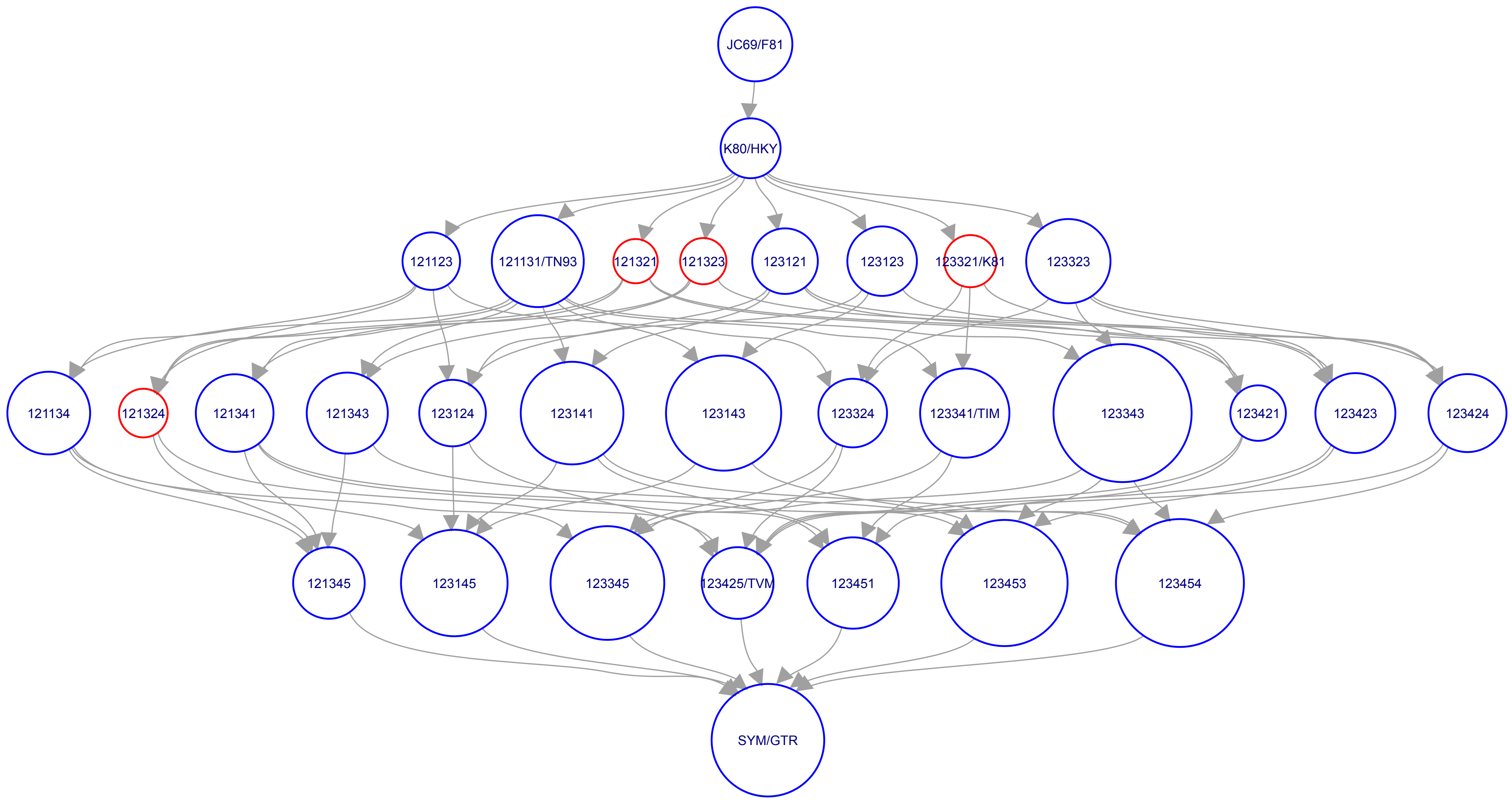

Supplement: S7 Fig — S5 Fig represents the posterior support of each of the models included in the search space. The size of the models’ bubble is proportional to its posterior support. Model bubbles with a blue outline are inside the 95% HPD. Model bubbles with a red outline have at most 0.27% support. (PDF) [file pone.0349373.s008.pdf]
